# Supplementary figures and images for: Evidence for a Role of Extraintestinal Pathogenic Escherichia coli , Enterococcus faecalis and Streptococcus gallolyticus in the Aetiology of Exudative Cloacitis in the Critically Endangered Kākāpō ( Strigops habroptilus )
Source: Mol Ecol. 2025 Apr 19;34(24):e17761. doi: 10.1111/mec.17761 (PMC12717991; doi:10.1111/mec.17761)

a. Ormycovirus

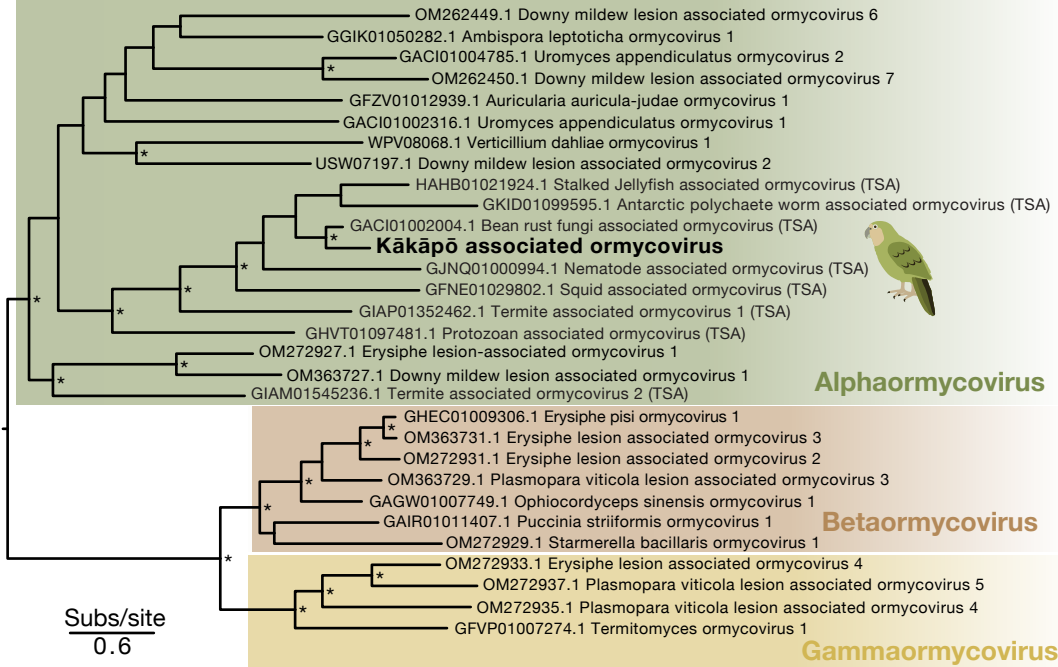

b.

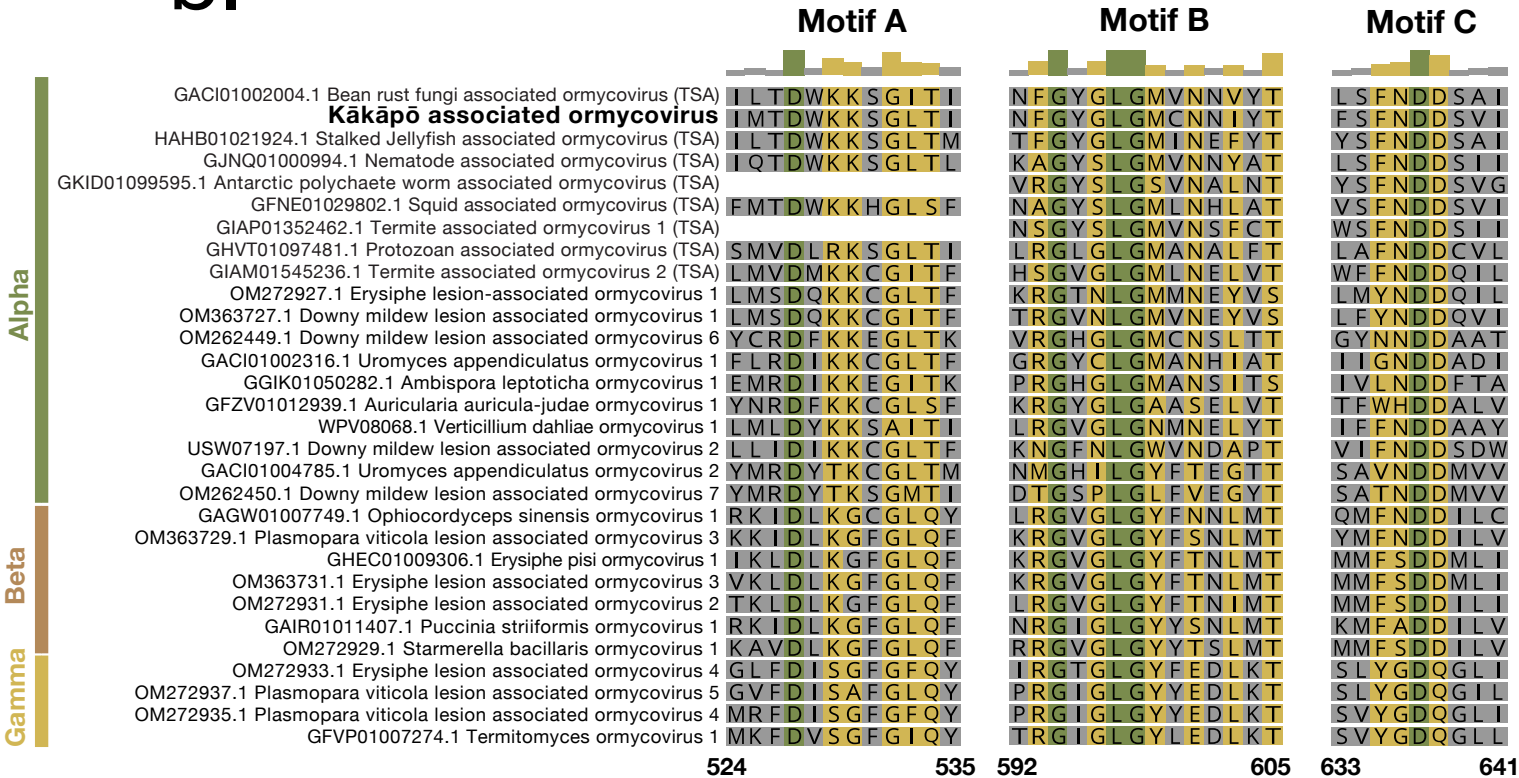

Supplement: Supplementary file 1 — Figure S1. Ormycovirus phylogeny and conserved motifs. Maximum likelihood midpoint rooted phylogenetic tree (a) of representative ormycovirus transcripts containing the RdRp. The kākāpō associated ormycovirus identified in this study is bolded while viruses that were identified through screening the TSA are noted. Branches are scaled to the number of amino acid substritutions per site. Nodes with ultrafast bootstrap values of > 90% are noted by an asterisk. Below the phylogeny is an alignment of ormycovirus RdRp amino acid sequences (b). Conserved motifs (A–C) are shown while the kākāpō associated ormycovirus is bolded. [file MEC-34-e17761-s004.pdf]

(A) *aroE*

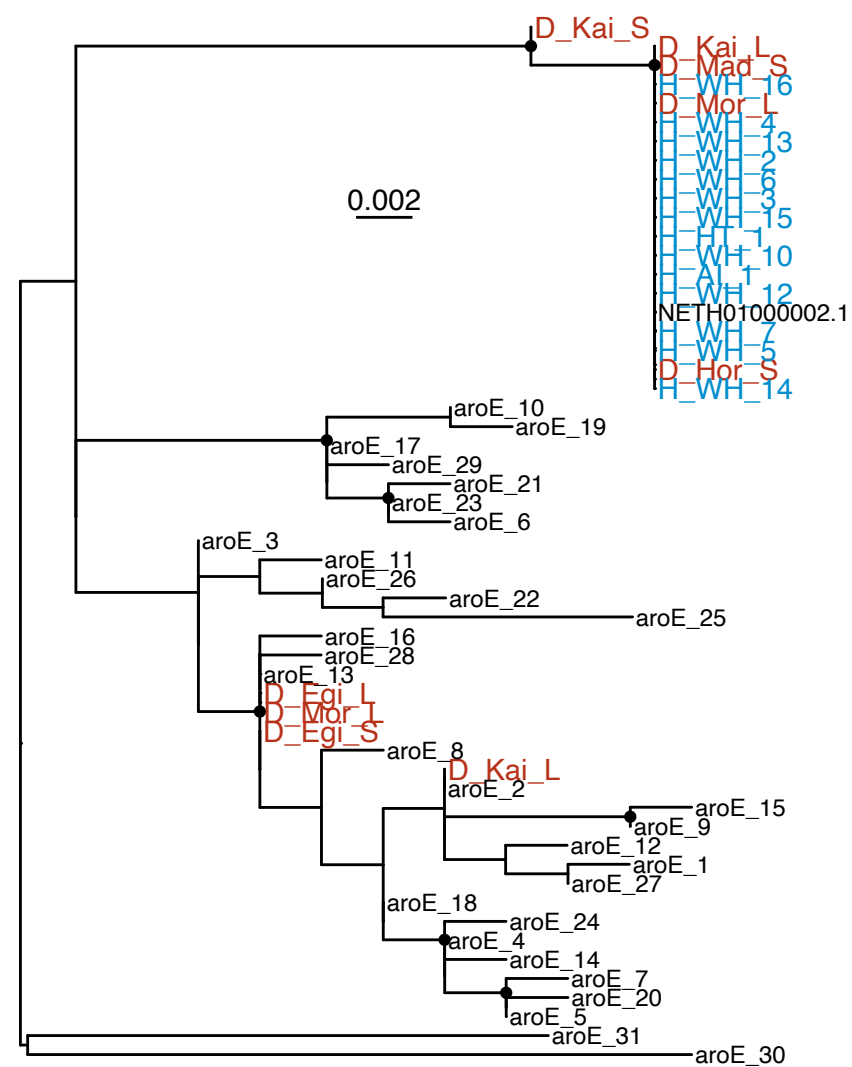

(C) *TrpD*

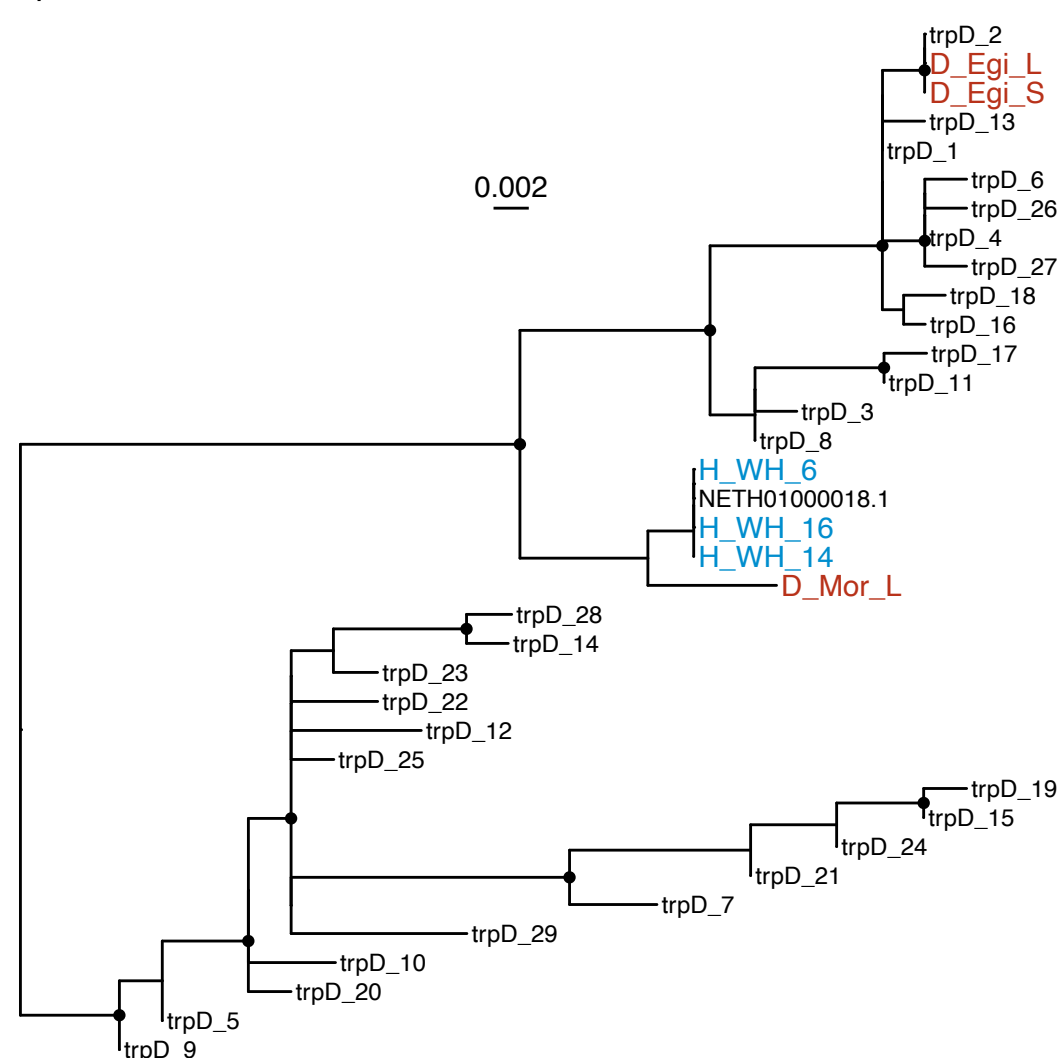

(E) *uvrA*

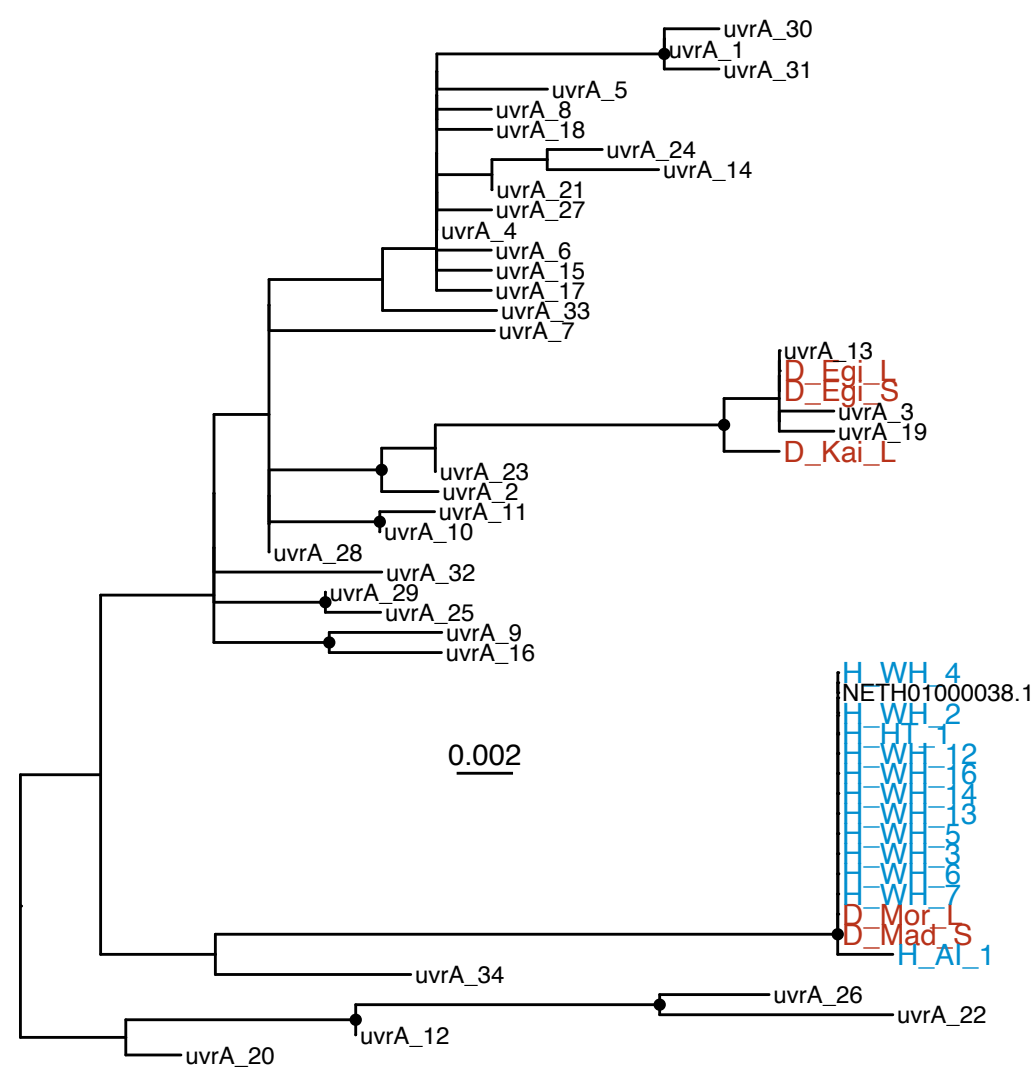

(G) *glgB*

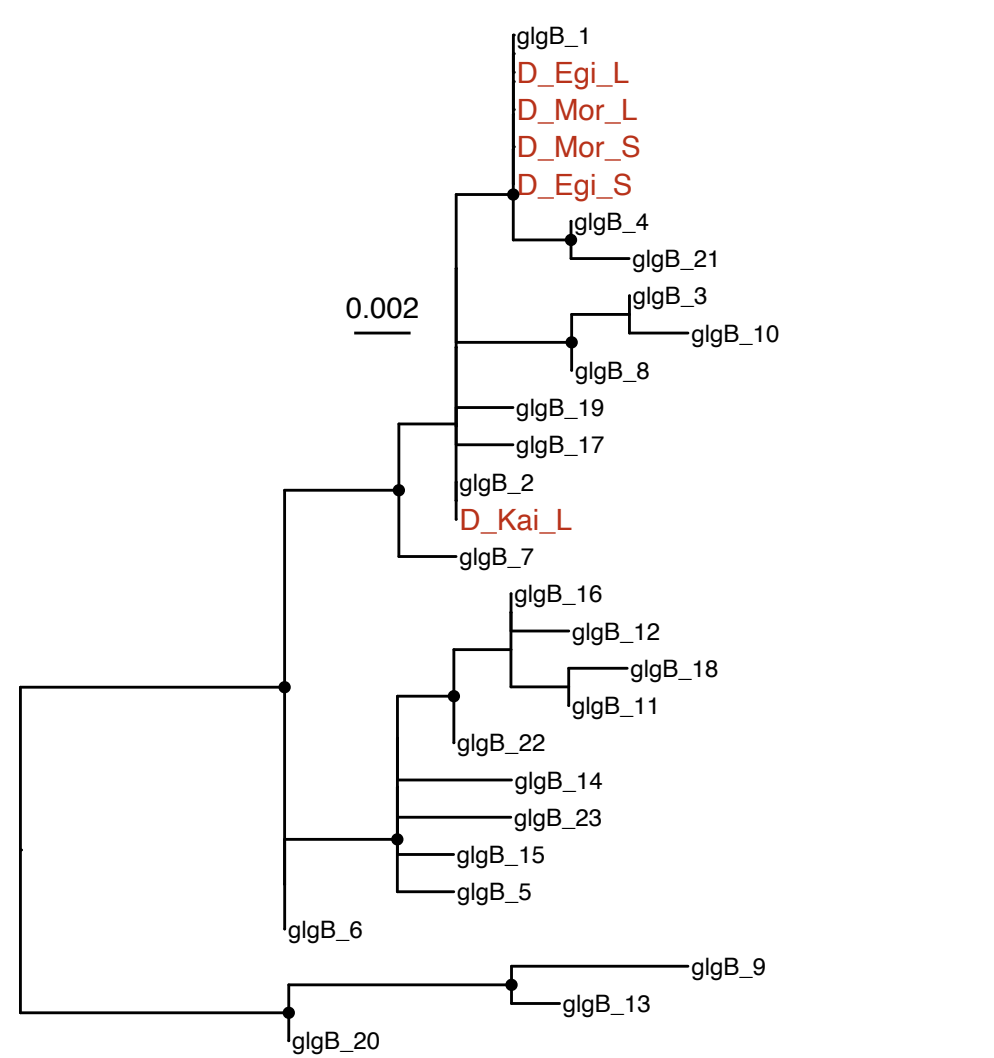

(B) *nifS*

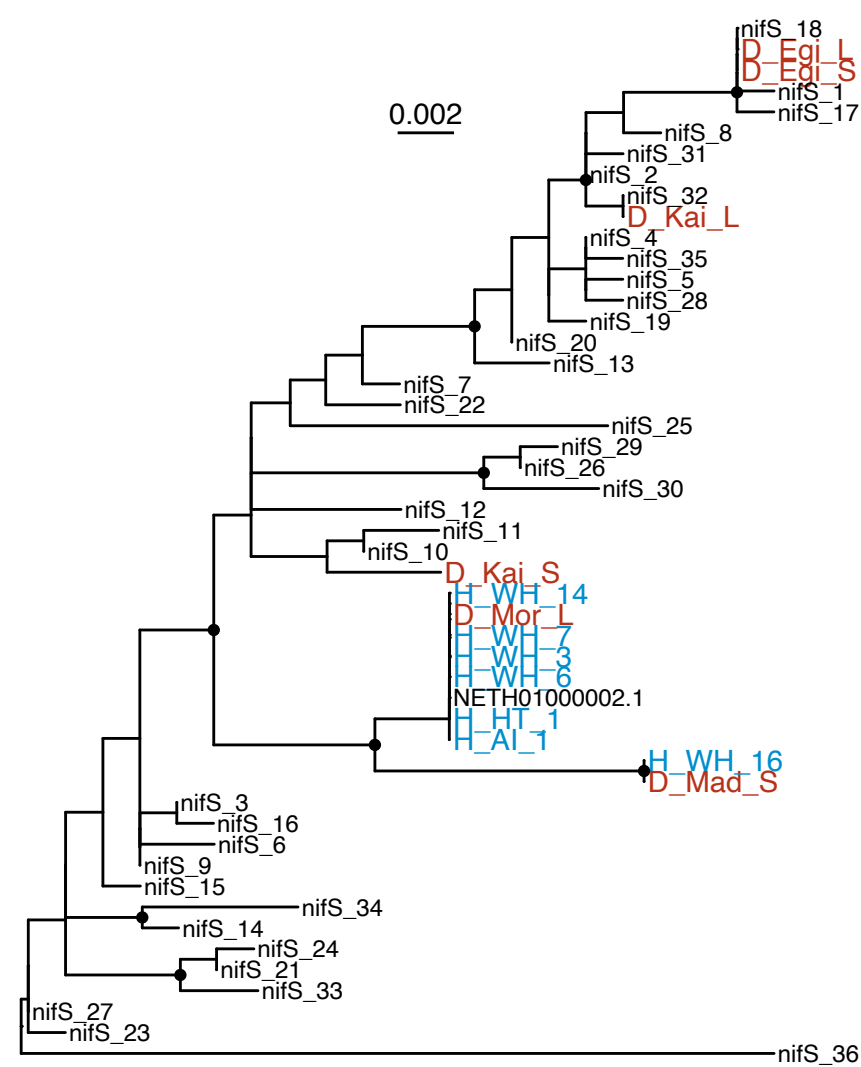

(D) *p20*

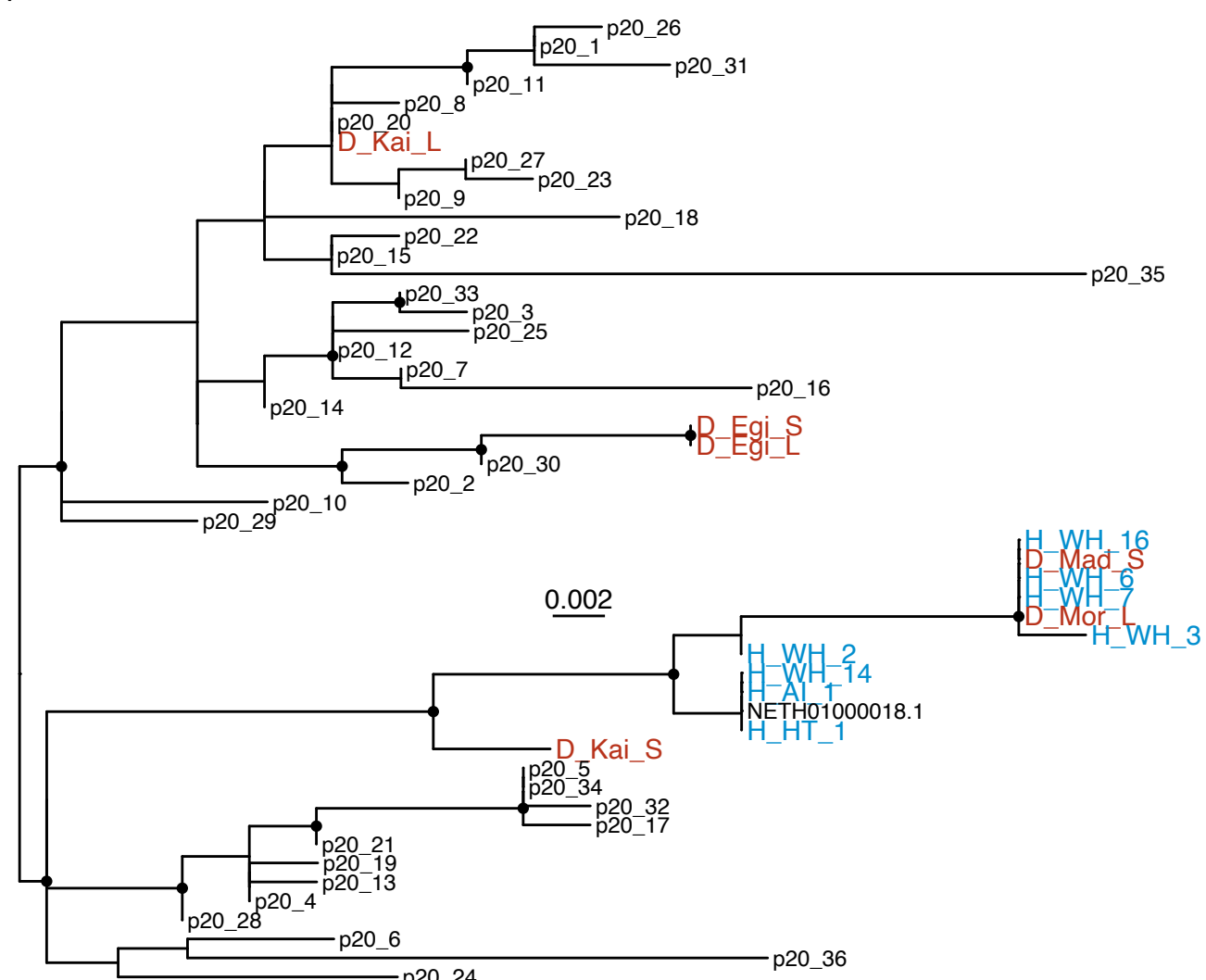

(F) *tk*t

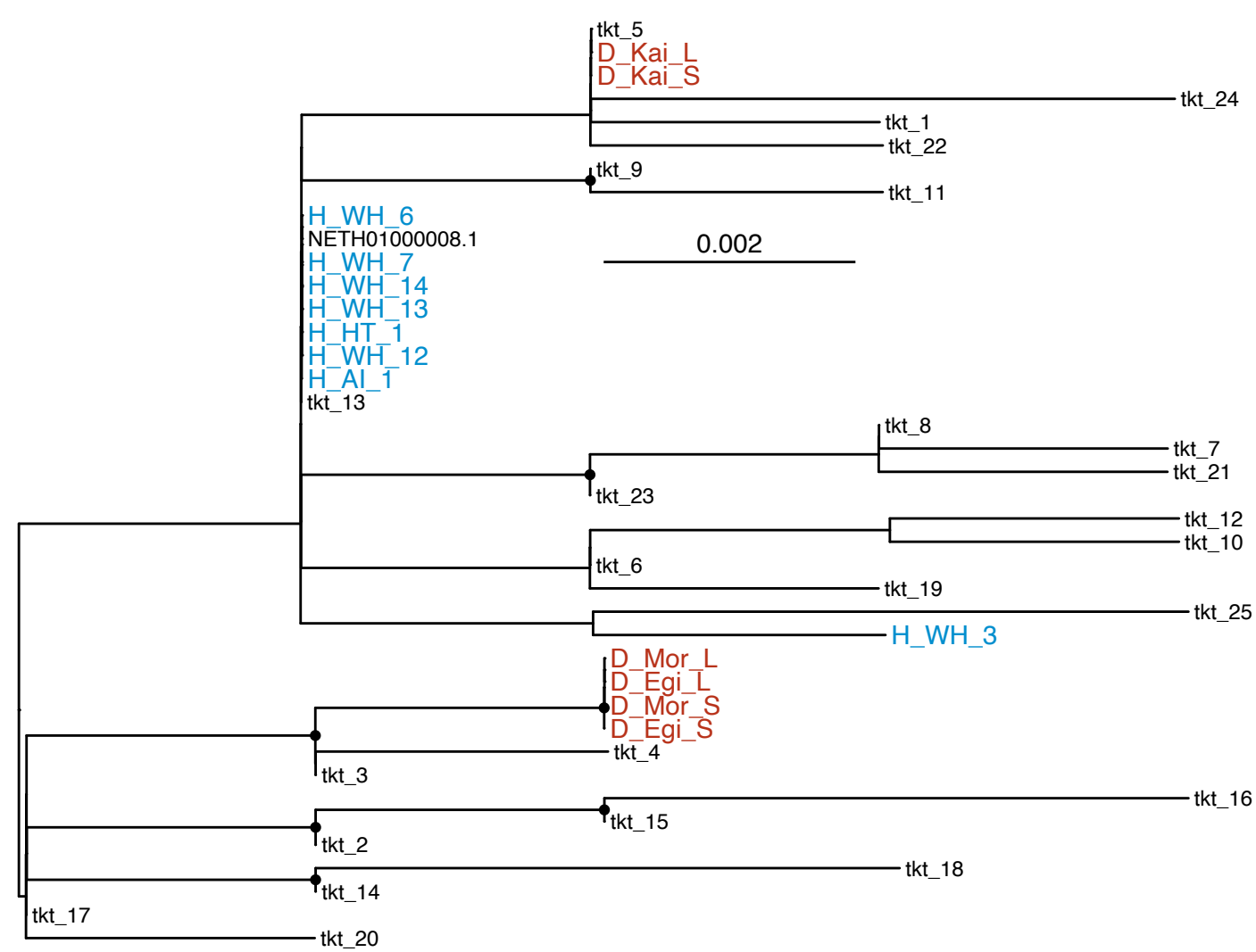

Supplement: Supplementary file 2 — Figure S2. Maximum likelihood phylogenetic tree of the seven Streptococcus gallolyticus loci used in multi‐locus sequence typing (MLST). Sequences found in healthy libraries are shown as blue labels, sequences found in diseased libraries shown in red. Alleles from the PubMLST database are shown in black. The sequences extracted from a previous kākāpō shotgun metagenomic project (Waite et al. 2018), are also shown in black (NETH01000002.1—NETH01000038.1). Branches are scaled according to the number of amino acid substitutions per site, shown in the scale bars. The trees are midpoint rooted for display purposes only. Black circles on nodes show bootstrap support values of more than 85%. [file MEC-34-e17761-s008.pdf]
