# Supplementary material for: Evidence for a Role of Extraintestinal Pathogenic Escherichia coli , Enterococcus faecalis and Streptococcus gallolyticus in the Aetiology of Exudative Cloacitis in the Critically Endangered Kākāpō ( Strigops habroptilus )
Source: Mol Ecol. 2025 Apr 19;34(24):e17761. doi: 10.1111/mec.17761 (PMC12717991; doi:10.1111/mec.17761)
Supplement: Supplementary file 3 — Figure S3. Maximum likelihood phylogenetic tree of the seven Enterococcus faecalis loci used in multi‐locus sequence typing (MLST). Sequences found in diseased libraries shown in red. Alleles from the PubMLST database are shown in black. Black circles on nodes show bootstrap support values of more than 85%. [file MEC-34-e17761-s006.pdf]

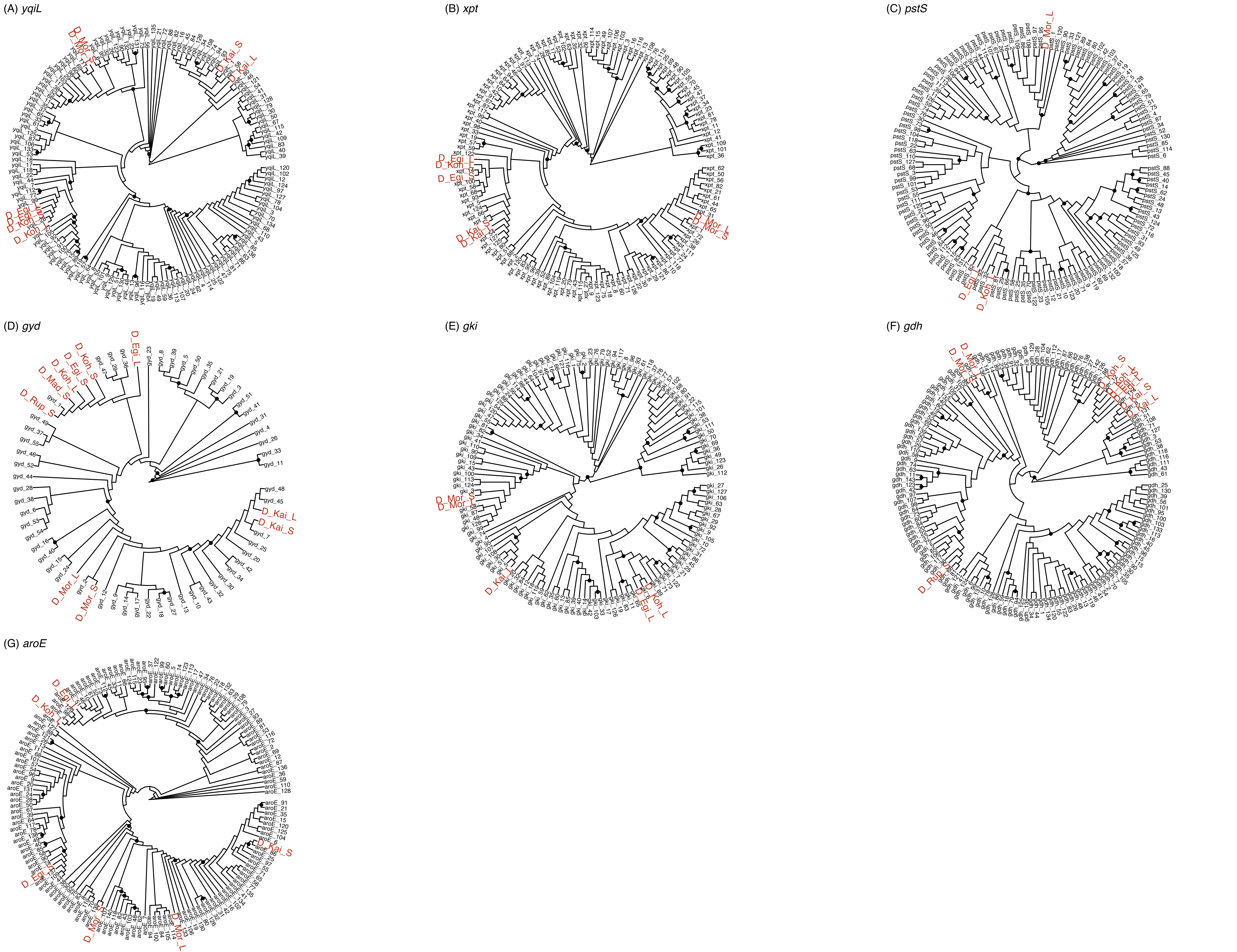
(A) *yqiL*
(B) *xpt*
(C) *pstS*
(D) *gyd*
(E) *gki*
(F) *gdh*
(G) *aroE*
